# The Efficiency of the Krebs Cycle and the Respiratory Chain in Physiologically and Prematurely Aging Bees (*Apis mellifera*)

**DOI:** 10.3390/ijms26157294

**Published:** 2025-07-28

**Authors:** Magdalena Kunat-Budzyńska, Patrycja Staniszewska, Krzysztof Olszewski, Małgorzata Cytryńska, Aneta Strachecka

**Affiliations:** 1 Department of Immunobiology, Institute of Biological Sciences, Faculty of Biology and Biotechnology, Maria Curie-Skłodowska University, Akademicka 19, 20-033 Lublin, Poland; magdalena.kunat-budzynska@mail.umcs.pl (M.K.-B.); malgorzata.cytrynska@mail.umcs.pl (M.C.); 2 Department of Invertebrate Ecophysiology and Experimental Biology, University of Life Sciences in Lublin, 20-950 Lublin, Poland; patrycja.staniszewska@up.lublin.pl; 3 Subdepartment of Apidology, Institute of Biological Basis of Animal Production, Faculty of Animal Sciences and Bioeconomy, University of Life Sciences in Lublin, 20-950 Lublin, Poland; krzysztof.olszewski@up.lublin.pl

**Keywords:** fat body, hemolymph, honeybee aging, respiratory chain, Krebs cycle, workers, *V. destructor*

## Abstract

The process of aging in organisms is associated with progressive metabolic changes that affect energy production. In our study, we compared the activities/concentrations of components related to the Krebs cycle and the respiratory chain (such as acetyl-CoA, IDH, AKG, succinate, fumarate, NADH_2_, UQCR, COX and ATP) in the hemolymph and fat body segments (tergites 3 and 5, sternite) in naturally and prematurely (affected by *V. destructor*) aging workers. Tergite 3 showed the highest metabolic activity, indicating its key role in energy storage and production. In naturally aging workers, the concentrations/activities of the tested components were higher in all the segments of the fat body and all the age groups when compared to the prematurely aging workers. The concentrations/activities of these components increased with age, usually reaching the maximum at 28 days of age in the fat body segments of naturally aging workers, and then decreasing in the oldest ones (at 35 days of age). An analysis of changes in the metabolic processes can provide a lot of important information on the mechanisms of aging. In the future, such studies can contribute to the development of effective strategies to delay the aging processes and improve the overall condition of bee colonies.

## 1. Introduction

Aging is a complex process influenced by various factors. Recent studies suggest that the main factors influencing the aging process are primarily the oxidative stress, gene expression, hormonal signals, as well as metabolic processes and energy metabolism [1]. Aging is characterized by chronic systemic inflammation, accompanied by cellular senescence, immunosenescence and organ dysfunction. Various anthropogenic and environmental factors (e.g., pathogens, mites, pesticides, environmental pollutants) promote chronic inflammation, which leads to immunopathology and, consequently, accelerated (or even premature) aging. At the same time, chronic inflammation accelerates the aging of immune cells, leading to weakened immune function and an inability to clear senescent cells and inflammatory factors, creating a vicious cycle of inflammation and aging [2,3,4].

Energy metabolism consists of many processes and mechanisms that are complex and still not fully understood. With age, a number of changes occur, including a decrease in the efficiency of the processes responsible for obtaining and using energy, which in consequence may affect health, susceptibility to diseases and the ability to regenerate. Therefore, it is important to understand the interrelationships between the aging process and the processes of obtaining and using energy, which can provide a lot of important information on the mechanisms of longevity, protection against cellular stress or physiological processes occurring in both humans and animals.

An excellent research model for studying changes in energy metabolism is insects, including honeybees. Honeybees have one of the highest rates of metabolism among animals. The metabolic rate is a key factor that determines how the body functions, how much energy it has and how it uses it, which is important in the context of the research on aging processes. The main components for energy production in bees are glucose and trehalose, where trehalose is produced in the fat body and can be released into the hemolymph where it is converted to glucose to satisfy the demand for energy [5]. The energy needed for thermoregulation and flight is produced in mitochondria during the Krebs cycle and oxidative phosphorylation. Bees achieve the highest level of energy efficiency during flight, showing a 100-fold increase in metabolism compared to those that stay inside the hive and have not flown yet [1,6,7,8,9]. As a result of intensive metabolic activity, oxidative stress occurs, which contributes to the aging of the organism. Our previous studies [10] have shown that the activity of antioxidant enzymes (catalase, glutathione-S-transferase, glutathione peroxidase, superoxidase dismutase) decreases with age and is lower in prematurely aging workers (affected by *V. destructor*).

The available literature provides information on the expression of genes-encoding enzymes in relation to the energy metabolism, but there is no information on the concentrations/activities of these enzymes [11,12,13,14,15,16,17]. High or low gene expression does not always indicate high or low activities of the tested enzymes, because the efficiency of their action is determined by various factors, e.g., environmental conditions or substrate availability.

Recently, in a study of the aging processes, changes occurring in the fat body have been analyzed on the strength of, among other things, the fact that the fat body in bees performs a function analogous to the human liver and adipose tissue and takes part in many metabolic processes. Furthermore, fat body cells do not show proliferative activity in adult bees [18,19,20,21]. Interestingly, lower rates of activity of mechanisms regulating energy management, energy consumption and metabolic changes were observed to correlate with age in workers, while such changes were not observed in queens, remaining at a similar level in them. The most numerous cells that build the fat body are trophocytes, which contain numerous lipid droplets in their cytoplasm. The main function of trophocytes is the accumulation of nutrients that are necessary for energy production and consumption. These reserve substances are stored in the form of glycogen and triglycerides, which are broken down into glucose or fatty acids. These then serve as an energy source for bees in various conditions [5,22,23]. The second most numerous cells in the fat body are oenocytes, whose key function is carbohydrate synthesis [18,19,20,21,24,25]. Chuang and Hsu [26] and Hsu and Chuang [27] showed that young workers have higher levels of ATP and NAD^+^ and a higher NAD^+^/NADH ratio in trophocytes than foragers, whose metabolic activity is reduced. We decided to supplement these studies with determinations of concentrations/activities of a larger number of Krebs cycle and respiratory chain compounds, not only in the hemolymph and in the entire fat body (without specifying the location: visceral vs. subcuticular), but above all in individual segments of the subcuticular fat body [26,27,28]. The fat body segments, i.e., tergites 3 and 5 and the sternite, were selected for the study due to their highest metabolic activity. For example, the sternite fat body, composed mainly of oenocytes, is characterized by a high protein content, providing amino acids that are converted into Krebs cycle intermediates, such as acetyl-CoA. In contrast, the fat body from the third tergite, consisting predominantly of trophocytes, has high levels of glucose, glycogen and lipids—energy substrates essential for ATP production. The fat body from the fifth tergite is the “bee liver” and is responsible for detoxification and proteolytic reactions [29,30,31].

One of the reasons causing the previously mentioned early inflammation and premature aging in honeybees is infestation by the *V. destructor* mite [32]. This mite has an inefficient metabolism and requires large amounts of nutrients, which it obtains by sucking the bees’ hemolymph and feeding on their fat body [33,34]. According to the literature, *V. destructor* infestation affects bee metabolism. Aldea and Bozinovic [35] demonstrated that the energy expenditure of a bee infested with two mites is double that of a healthy bee. This causes the bees to tire more quickly because they use up more energy, which accelerates the aging process and consequently leads to their death.

Therefore, we hypothesized that *V. destructor*, which accelerates the aging processes, affects the energy metabolism of bees. Prematurely aging workers (affected by *V. destructor*) exhibit lower concentrations/activities of the Krebs cycle components [acetyl coenzyme A (acetyl-CoA), isocitrate dehydrogenase (IDH), alpha-ketoglutarate (AKG), succinate, fumarate, nicotinamide adenine dinucleotide (NADH_2_)], lower activities of the respiratory chain enzymes [cytochrome c reductase (UQCR), cytochrome c oxidase (COX)], as well as lower levels of adenosine triphosphate (ATP)] compared to naturally (physiologically) aging workers.

The aim of our study was to evaluate the efficiency of the Krebs cycle and the respiratory chain in the hemolymph and fat body segments between naturally (physiologically) aging workers and prematurely aging ones (affected by *V. destructor)*. Our research will supplement the existing knowledge of the concentrations/activities of compounds involved in the processes of energy acquisition, using the example of the Krebs cycle and the respiratory chain in naturally (physiologically) and prematurely aging workers (affected by *V. destructor*).

## 2. Results

The tissue location (hemolymph or fat body from different segments—tergite 3, tergite 5, sternite) had a statistically significant effect on the compound levels in the naturally (physiologically) and prematurely aging workers (Table S1).

The concentrations of acetyl-CoA, AKG, succinate, fumarate, NADH_2_, ATP and the activities of COX and UQCR and IDH differed statistically significantly depending on the age of the workers (1, 14, 21, 28 and 35 days) (Table S2).

**Figure 1:**
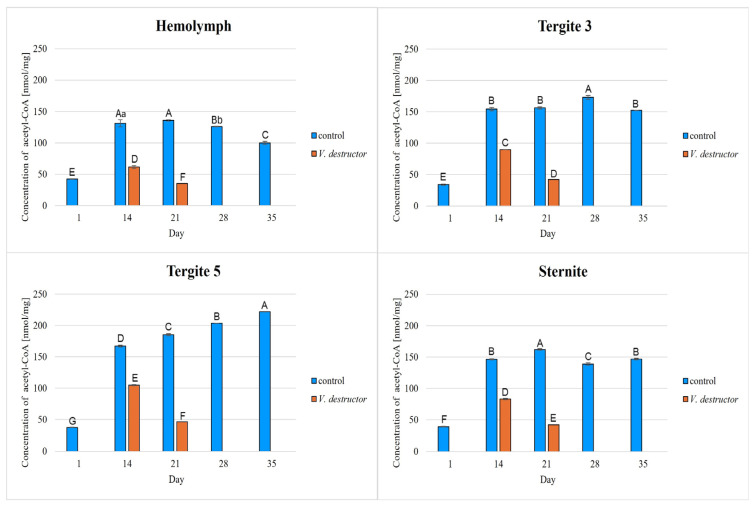
Acetyl coenzyme A (acetyl-CoA) concentrations [nmol/mg tissues] in the hemolymph and the fat body from tergite 3, tergite 5 and the sternite of the naturally (physiologically) aging 1-, 14-, 21-, 28-, and 35-day-old workers and in the prematurely aging 14- and 21-day-old workers (affected by *V. destructor*). A–G—capital letters indicate statistically significant differences between the groups at *p* ≤ 0.01. a, b—lowercase letters indicate statistically significant differences between the groups at *p* ≤ 0.05. Bars indicate standard deviation.

**Figure 2:**
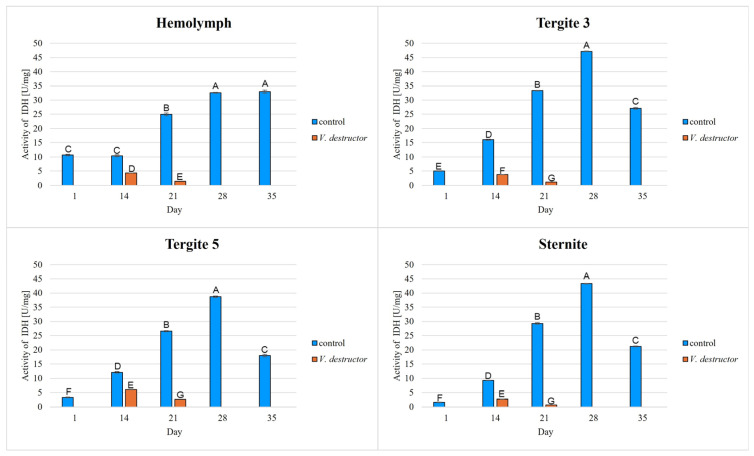
Isocitrate dehydrogenase (IDH) activities [U/mg protein] in the hemolymph and the fat body from tergite 3, tergite 5 and the sternite of the naturally (physiologically) aging 1-, 14-, 21-, 28- and 35-day-old workers and in the prematurely aging 14- and 21-day-old workers (affected by *V. destructor*). A–G—capital letters indicate statistically significant differences between the groups at *p* ≤ 0.01. Bars indicate standard deviation.

**Figure 3:**
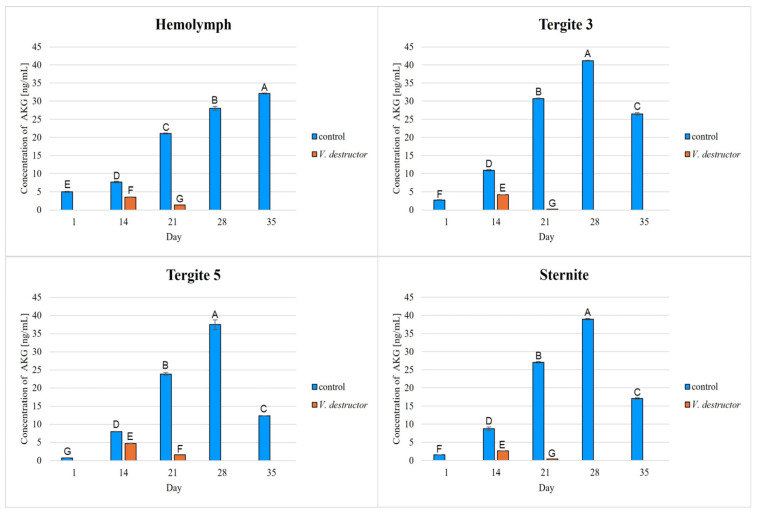
Alpha-ketoglutarate (AKG) concentrations in the hemolymph and the fat body from tergite 3, tergite 5 and the sternite of the naturally (physiologically) aging 1-, 14-, 21-, 28- and 35-day-old workers and in the prematurely aging 14- and 21-day-old workers (affected by *V. destructor*). A–G—capital letters indicate statistically significant differences between the groups at *p* ≤ 0.01. Bars indicate standard deviation.

**Figure 4:**
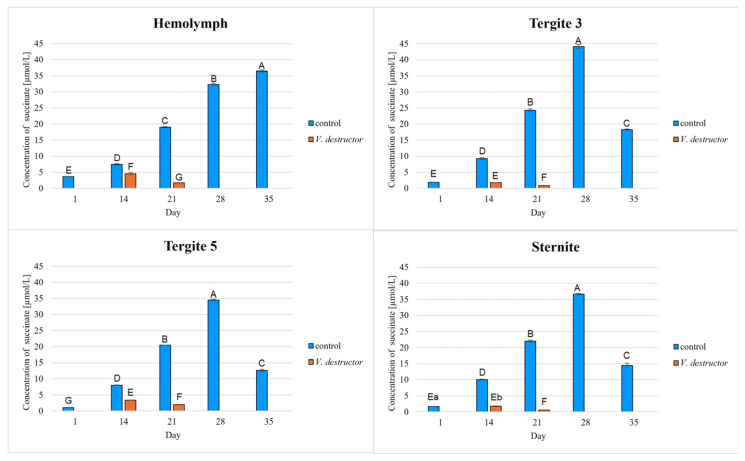
Succinate concentrations in the hemolymph and the fat body from tergite 3, tergite 5 and the sternite of the naturally (physiologically) aging 1-, 14-, 21-, 28- and 35-day-old workers and in the prematurely aging 14- and 21-day-old workers (affected by *V. destructor*). A–G—capital letters indicate statistically significant differences between the groups at *p* ≤ 0.01. a, b—lowercase letters indicate statistically significant differences between the groups at *p* ≤ 0.05. Bars indicate standard deviation.

**Figure 5:**
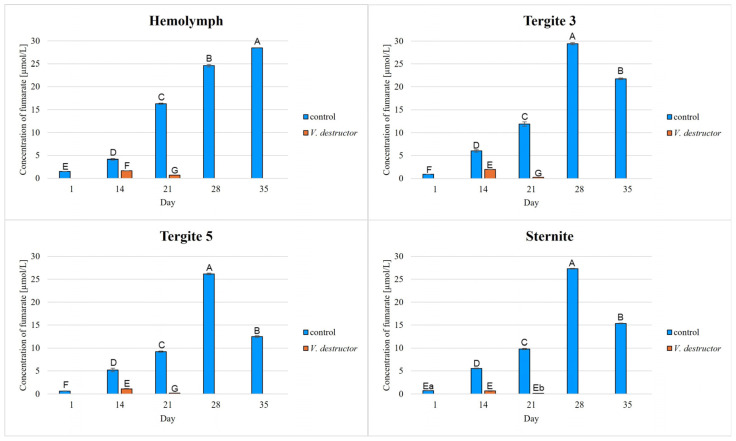
Fumarate concentrations in the hemolymph and the fat body from tergite 3, tergite 5 and the sternite of the naturally (physiologically) aging 1-, 14-, 21-, 28- and 35-day-old workers and in the prematurely aging 14- and 21-day-old workers (affected by *V. destructor*). A–G—capital letters indicate statistically significant differences between the groups at *p* ≤ 0.01. a, b—lowercase letters indicate statistically significant differences between the groups at *p* ≤ 0.05. Bars indicate standard deviation.

**Figure 6:**
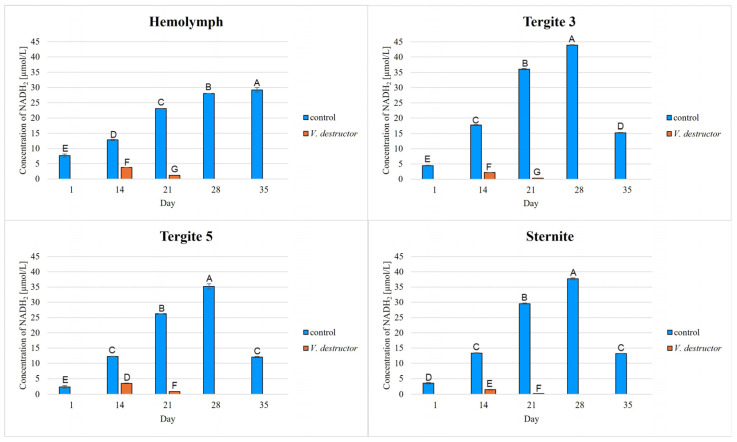
Nicotinamide adenine dinucleotide (NADH_2_) concentrations in the hemolymph and the fat body from tergite 3, tergite 5 and the sternite of the naturally (physiologically) aging 1-, 14-, 21-, 28- and 35-day-old workers and in the prematurely aging 14- and 21-day-old workers (affected by *V. destructor*). A–G—capital letters indicate statistically significant differences between the groups at *p* ≤ 0.01. Bars indicate standard deviation.

**Figure 7:**
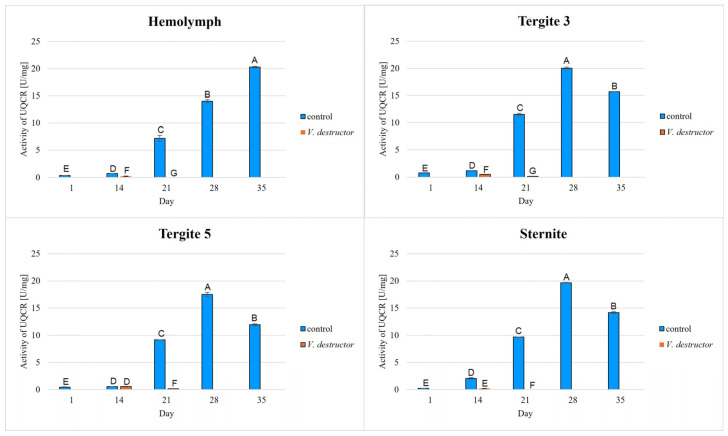
Cytochrome c reductase (UQCR) activities [U/mg protein] in the hemolymph and the fat body from tergite 3, tergite 5 and the sternite of the naturally (physiologically) aging 1-, 14-, 21-, 28- and 35-day-old workers and in the prematurely aging 14- and 21-day-old workers (affected by *V. destructor*). A–G—capital letters indicate statistically significant differences between the groups at *p* ≤ 0.01. Bars indicate standard deviation.

**Figure 8:**
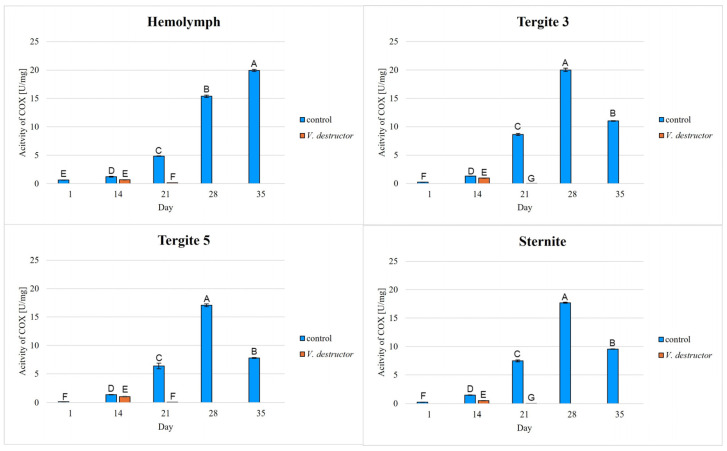
Cytochrome c oxidase (COX) activities [U/mg protein] in the hemolymph and the fat body from tergite 3, tergite 5 and sternite of the naturally (physiologically) aging 1-, 14-, 21-, 28- and 35-day-old workers and in the prematurely aging 14- and 21-day-old workers (affected by *V. destructor*). A–G—capital letters indicate statistically significant differences between the groups at *p* ≤ 0.01. Bars indicate standard deviation.

**Figure 9:**
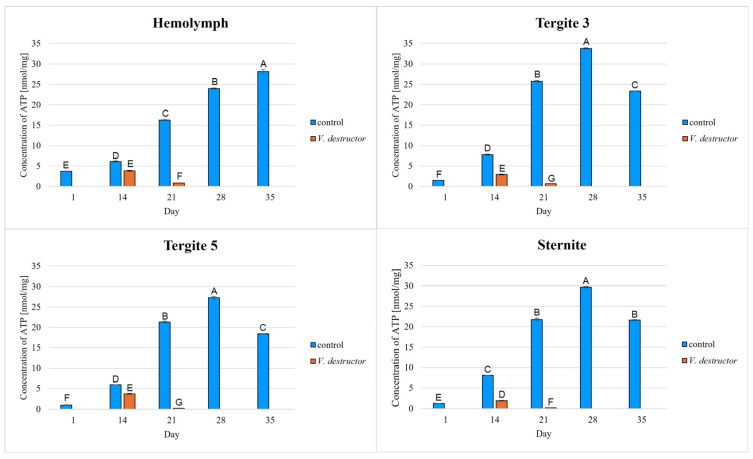
Adenosine triphosphate (ATP) concentrations in the hemolymph and the fat body from tergite 3, tergite 5 and the sternite of the naturally (physiologically) aging 1-, 14-, 21-, 28- and 35-day-old workers and in the prematurely aging 14- and 21-day-old workers (affected by *V. destructor*). A–G—capital letters indicate statistically significant differences between the groups at *p* ≤ 0.01. Bars indicate standard deviation.

The highest concentrations of AKG, succinate, fumarate, NADH_2_, ATP, as well as the highest IDH, COX and UQCR activities were noted in the fat body from tergite 3, while for acetyl-CoA the highest concentrations were observed in the fat body from tergite 5 in the naturally (physiologically) aging workers (Figure 1, Figure 2, Figure 3, Figure 4, Figure 5, Figure 6, Figure 7, Figure 8 and Figure 9).

The concentrations of AKG, succinate, fumarate, NADH_2_, ATP, as well as the IDH, COX and UQCR activities increased in the fat body from tergite 3 and 5 and the sternite with age, reaching a maximum in the naturally (physiologically) aging workers at 28 days of age, and then decreased in the oldest (35-day-old) workers. In the hemolymph, the concentrations and activities of the above-mentioned compounds increased with age reaching a maximum at 35 days of age (Figure 2, Figure 3, Figure 4, Figure 5, Figure 6, Figure 7, Figure 8 and Figure 9). The highest concentrations of acetyl-CoA were observed in the following: in the hemolymph and fat body from the sternite in the 21-day-old workers, in the fat body from tergite 3 in the 28-day-old workers and in the fat body from tergite 5 in the 35-day-old naturally (physiologically) aging workers (Figure 1).

The concentrations of acetyl-CoA, AKG, succinate, fumarate, NADH_2_, ATP, and IDH, COX and UQCR activities in the naturally (physiologically) aging workers were statistically significantly higher in all the fat body tissues/locations and age groups compared to prematurely aging workers (affected by *V. destructor*) (Figure 1, Figure 2, Figure 3, Figure 4, Figure 5, Figure 6, Figure 7, Figure 8 and Figure 9). An exception was UQCR activity in the fat body from tergite 5, where the activity was slightly higher in the 14-day-old prematurely aging workers compared to naturally (physiologically) aging workers (Figure 7). In the prematurely aging workers, the concentrations of acetyl-CoA, AKG, succinate, fumarate, NADH_2_ and ATP, and the IDH, COX and UQCR activities reached a maximum in the 14-day-old workers and were significantly higher compared to the 21-day-old workers (Figure 1, Figure 2, Figure 3, Figure 4, Figure 5, Figure 6, Figure 7, Figure 8 and Figure 9).

## 3. Discussion

The main source of energy for cells is ATP, which is produced in the cytoplasm of the cell in the process of glycolysis and then in the mitochondria in the processes of the Krebs cycle and oxidative phosphorylation [36,37].

It has been documented that two intermediary components of the Krebs cycle, i.e., alpha-ketoglutarate and succinate, play an important role in the aging process. In addition to their role in energy metabolism, these components also influence important cellular processes such as amino acid synthesis, gene control, cell differentiation and cancer development [38]. In addition, the role of alpha-ketoglutarate has been studied in various models, including mice, nematodes and *Drosophila melanogaster* in the context of aging processes. AKG has antioxidant properties, inhibits cancer development and controls energy production [39].

In our work, we took into account the fundamental role played by the Krebs cycle and the respiratory chain in regulating energy metabolism, which in turn may have a direct impact on the aging processes in various organisms, including bees. Bees are an interesting research model in the context of aging processes because there is clear evidence of relationships between energy metabolism and their lifespan. For example, bees have a division of labor, i.e., into younger hive bees, whose main task is to care for the larvae, and older forager bees, which are responsible for working outside the hive. The transition of a bee from a nurse to a forager is associated with many changes in its metabolism and physiology. Foragers feed during flights mainly on pollen and nectar, which contain predominantly simple sugars. Such a diet, combined with intense exercise during the flight, can lead to increased energy consumption in comparison with the nurses, whose diet is more diverse. A change in the diet can affect the composition of the gut microbiome, impaired nutritional conditions, differential gene expression and increased protein oxidation processes. Consequently, such changes accelerate the aging process [1,40,41,42].

### 3.1. The Role of Age and Tissue/Locations of the Fat Body in Changes in Energy Metabolism

Various factors and compounds, such as pesticides or the magnetic field, can accelerate the aging process and influence the tendency of energy changes in bee tissues [17,28,43]. Paleolog et al. [28] noted that the activities/concentrations of the respiratory chain and the Krebs cycle compounds (acetyl-CoA, isocitrate dehydrogenase, AKG, succinate, fumarate, NADH_2_, UQCR, COX, and ATP) are higher in the fat body compared to the hemolymph in worker bees. Imidacloprid administered in two doses of 200 ppb and 5 ppb led to a decrease in the concentrations of the main energy carriers, i.e., ATP, NADH_2_ and acetyl-CoA. In addition, it was observed that at a higher concentration of imidacloprid there was a decrease in the concentrations of AKG, fumarate and succinate, while at a lower dose the opposite trend was observed, which may indicate a phenomenon called hormesis, i.e., a defensive reaction to a low dose/stress. However, these authors treated the fat body as a homogeneous tissue, specifying only that it was collected from the tergites between the third and fifth abdominal segments. Our study refines this information and shows the concentrations/activities of these compounds in individual fat body segments from the third tergite, fifth tergite and sternite indicating considerable differences between these fat body segments. In turn, Migdał et al. [43] showed that, by reducing the activities of enzymatic biomarkers (aspartate aminotransferase—AST, alanine aminotransferase—ALT and alkaline phosphatase—ALP), the magnetic field can also disrupt key metabolic processes responsible for energy production, including the Krebs cycle, oxidative phosphorylation or ATP synthesis. Additionally, Murawska et al. [17] showed reduced expression of the COX5a enzyme (involved in oxidative phosphorylation) in 7-day-old bees exposed to electric fields, which consequently led to faster aging [17].

The influence of various substances that improve metabolic activity in bees has also been studied. Burzyński et al. [11] showed increased mRNA expression of enzymes related to the Krebs cycle in bees’ heads, including isocitrate dehydrogenase and succinate dehydrogenase, with the exception of the alpha-ketoglutarate dehydrogenase (down-regulation) after the administration of phenylbutyrate and phenylacetylglutamate. An important phenomenon is that with the increase in the activity of the Krebs cycle, glucose metabolism in neurons increases, which in turn has a positive effect on the brain function. Increased metabolic activity after the administration of the above-mentioned compounds is also associated with enhanced antioxidant protection, which consequently inhibits the aging process by removing free radicals responsible for cell damage.

To date, most studies have focused on the analysis of ATPase activities or ATP concentrations in whole bees or in the entire fat body or in the hemolymph of bees [26,27,44]. Our studies specify and complement these reports with the analysis of concentrations/activities of as many as nine key components participating in the Krebs cycle and the respiratory chain. An innovative approach is to analyze these compounds not in the entire tissue, but in specific segments/locations of the fat body and in the hemolymph. Hsu and Chuang [27] found higher ATP concentrations in the trophocytes of young bees in comparison with foragers. We observed a similar trend in our study, where the ATP concentrations increased with age, reaching a maximum in the 28-day-old naturally (physiologically) aging workers, and then decreasing in the 35-day-old workers in the fat body from tergite 3 and 5 and the sternite (Figure 9). In addition, our studies elaborated this information by determining the changes in the activities/concentrations of these nine compounds in bees along with their aging processes, taking into account natural (physiological) and accelerated aging.

In the analysis of the functions of the honeybee fat body, we focused on its segmented structure. The individual fat body segments, i.e., tergites 3 and 5 and the sternite, play separate functional roles, which may be important for the metabolic and physiological differences between the different stages in bee life [23]. Strachecka et al. [23] and Bryś et al. [29] showed that the largest production and storage of energy compounds, i.e., glucose, glycogen and triglycerides, take place in tergite 3, which is composed mainly of trophocytes. These compounds are essential substrates for energy production in the Krebs cycle and oxidative phosphorylation. The location of tergite 3 is also important, because it is situated close to the heart and body cavities, which allows for a rapid distribution of components to tissues via the circulatory system [29]. This is consistent with our results as we have shown for the first time that the highest concentrations/activities were reached by the Krebs cycle and respiratory chain components in tergite 3 in both the naturally and prematurely aging bees (Figure 1, Figure 2, Figure 3, Figure 4, Figure 5, Figure 6, Figure 7, Figure 8 and Figure 9).

In our studies, we have shown the highest concentrations/activities of the Krebs cycle and respiratory chain components in the fat body segments in the 21- and 28-day-old bees, which are physiologically foragers in the bee colony. This indicates high energy metabolism in such naturally aging foragers (21 and 28-day-old) (Figure 1, Figure 2, Figure 3, Figure 4, Figure 5, Figure 6, Figure 7, Figure 8 and Figure 9). At the same time, bees of this age are characterized by high antioxidant (catalase, glutathione-S-transferase, glutathione peroxidase, superoxidase dismutase) activities in the particular fat body segments and in the hemolymph [10]. This is partly consistent with the results obtained by Menail et al. [1] who observed an age-related increase in the activities of malate dehydrogenase (a Krebs cycle enzyme) and NADH dehydrogenase (an enzyme in the respiratory chain) in the muscles of bees. Cervoni et al. [41] noticed that the heads and abdomens of nurse bees have higher mitochondrial activity than those of foragers. They found the highest activity in the thorax to be connected with the flight muscles located there. During flight, metabolic activity increases, leading to faster energy consumption and, consequently, cell loss. At the same time, greater antioxidant activities are also observed. In turn, Schippers et al. [9] showed that in foragers, the enzyme activities related to cellular respiration, citrate synthase and cytochrome c oxidase, remained constant, but the metabolic rate increased.

In the worker bees, we have observed that there is an interaction between the hemolymph and the segments of the fat body, which is crucial for the regulation of energy metabolism. The hemolymph of bees contains metabolic compounds, such as glucose and amino acids, which can be absorbed by the fat body cells and transformed inside them into metabolites participating in the Krebs cycle. In the 35-day-old naturally (physiologically) aging workers, we found metabolic activity to be elevated in the hemolymph, while it decreased in the fat body (Figure 1, Figure 2, Figure 3, Figure 4, Figure 5, Figure 6, Figure 7, Figure 8 and Figure 9). This may indicate that with age, the main metabolic activity is transferred from the fat body to other tissues, for example the hemolymph, or the fat body gradually loses its metabolic function, which leads to a decrease in metabolic efficiency and accelerated aging.

### 3.2. The Role of V. destructor Infestation in Changes in Energy Metabolism

Another important issue that we addressed in our studies was the influence of the *V. destructor* parasite on the aging processes in the context of changes in energy metabolism. To date, it is known that by feeding on the fat body and hemolymph of bees [33,34], *V. destructor* reduces the amount of carbohydrates in the bees’ abdomen [45] and hemolymph [46]. This is consistent with our observations, in which we showed that prematurely aged bees infested with *V. destructor* had lower concentrations/activities of acetyl-CoA, IDH, AKG, succinate, fumarate, NADH_2_, ATP, COX and UQCR as compared to naturally (physiologically) aging workers (Figure 1, Figure 2, Figure 3, Figure 4, Figure 5, Figure 6, Figure 7, Figure 8 and Figure 9). As a result, energy production in the bees’ bodies decreased and they became weaker, which led to their premature death. The bees infested with *V. destructor* survived only 21 days in comparison with the naturally (physiologically) aging workers, which survived 35 days (Figure 1, Figure 2, Figure 3, Figure 4, Figure 5, Figure 6, Figure 7, Figure 8 and Figure 9). Łopieńska-Biernat et al. [47] detected many compounds in the chemical composition of *V. destructor*, mainly carbohydrates, as well as lipids and proteins which come from bee tissues and are necessary for these mites for proper growth, reproduction and survival [33,34]. Of these carbohydrates, glucose was the most abundant in the body of *V. destructor*, with smaller amounts of glycogen and trehalose, which are the basic substrates for energy production in the process of cellular respiration [47]. Studies showed that *V. destructor* infections combined with pesticides can cause adverse changes in honeybees. This synergy leads to a weakened immune response, which in turn makes bees more susceptible to various diseases, including *Varroa*. For example, combining *V. destructor* with the pesticide acetamiprid disrupts energy metabolism and damages the midgut, which in turn disrupts the physiological balance of the bees [48]. Exposure of *V. destructor* to chlorothalonil shortens the lifespan of bees, leads to structural changes in their adipose tissue and disrupts the expression of genes responsible for, among other things, detoxification or nutrient metabolism [49]. Furthermore, combining infestation with imidacloprid reduces the bees’ flight ability, limiting their ability to forage [50]. However, the aforementioned studies focused primarily on changes in gene expression; the literature lacks studies examining the effects of *V. destructor* in combination with other stressors on the activity of compounds in the Krebs cycle and respiratory chain. Our study fills this gap by providing new data on the mechanisms of accelerated aging in bees. In the future, it is worth examining the synergistic effects of multiple stressors, e.g., pesticides, food deficiency and pathogens, on the aging process of bees. A more detailed understanding of the many mechanisms, including energy metabolism, that contribute to accelerated aging in bees may allow the development of various strategies to delay the aging process in bees.

Another group of pathogens, microsporidia, which include *Nosema ceranae*, *Nosema apis* and *Nosema bombycis*, also cause energy stress in bees. Microsporidia are intracellular pathogens that feed on the host’s nutrients and also obtain energy from it, since they are unable to produce energy in the form of ATP due to the absence of mitochondria [51,52]. Bees infected with *N. ceranae* are more likely to feel hungry and lose energy due to infection, which exerts energy stress on them. As a result, bees undergo premature aging and an accelerated transition to the role of foragers occurs, which exposes the bees to various risks, e.g., they may come into contact with various environmental pollutants, such as pesticides or heavy metals [51,52]. Another species of microsporidia, i.e., *N. bombycis,* in silkworm embryo cells caused an increase in the concentrations of compounds involved in glycolysis and the Krebs cycle: oxaloacetate, alpha-ketoglutarate and citrate. This may indicate an activation of energy-acquisition processes in response to the infection. In addition, the concentration of the end product of cellular respiration, ATP, did not change in the host cells under the influence of the infection, consequently leaving the host’s vital processes, including energy metabolism, undisturbed and allowing them to survive the infection [53].

## 4. Materials and Methods

### 4.1. Collecting One-Day-Old Bees for Experiments

Bees for the experiments were obtained from the apiary of the University of Life Sciences in Lublin (51°22′ N, 22°63′ E), Poland. Eight healthy bee colonies (in Dadant beehives) were selected for the experiments. A queen-excluder comb-cage was used to house the queens for 12 h in confinement with one void comb for laying eggs in it. On the twentieth day after the combs were laid with eggs, they were transferred to an incubator, where one-day-old bees emerged. Six thousand one-day-old bees were color coded (POSCA PC-3M marker, Uni Mitsubishi Pencil, Shinagawa, Tokyo, Japan) and transferred to six colonies prepared in mini hives with small frames (210 mm × 170 mm), and thirty one-day-old bees were immediately collected for biochemical analysis. The aforementioned colonies were divided into two experimental groups, i.e., a naturally/physiologically aging control group (3 colonies) and a prematurely aging group infested with *V. destructor* (3 colonies). The colonies from the control groups were effectively treated against *Varroa*. The applied treatments consisted of isolating the queen to bring her to a brood-free state preventing *V. destructor* from rapidly multiplying and heavily infecting the colony. Such treatments were carried out in the summer in June and in the autumn in October. After obtaining a brood-free state, the colonies were sublimated with oxalic acid. In addition, during the collection of workers for laboratory analyses, individual insects were selected, on the body of which the presence of *V. destructor* was not observed. Worker bees from the control groups were collected on the 14th, 21st, 28th and 35th day of life (3 colonies × 4 samplings × 10 bees). In the *V. destructor* infested group, 14- and 21-day-old worker bees with mites visible on their bodies were collected (3 colonies × 2 samplings × 10 bees). Due to the shortened lifespan of the infested bees, 28- and 35-day-old worker bees could not be collected.

From each colony in the control and infested groups, 10 workers were collected in each sampling session to obtain a representative number of bees. A total of 210 workers were taken for the experiments.

### 4.2. Laboratory Analyses

#### 4.2.1. Hemolymph Collection

Hemolymph was collected from living workers using a glass capillary (20 µL; the ‘end to end’ type; without an anticoagulant; Medlab Products, Raszyn, Poland) placed between the third and fourth tergites on the abdomen [54]. The volume of hemolymph was determined individually for each capillary. The hemolymph collected from the individual bees in a volume of 5 µL was then placed in an Eppendorf tube containing 25 µL of ice-cold 0.6% NaCl. The obtained hemolymph solutions were stored at −40 °C until further biochemical analyses.

#### 4.2.2. Fat Body Collection

For biochemical analyses, the most metabolically active segments of the fat body, i.e., tergites 3 and 5 and sternites [23], were collected according to the methodology described by Bryś et al. [29]. The location of individual segments was shown in photos in our earlier publication [10]. Tissues were weighed at a maximum temperature of 4 °C. The tissues were manually homogenized and then centrifuged at 3000× *g* at 4 °C for 1 min. The obtained supernatants were stored at −25 °C until further biochemical analyses.

#### 4.2.3. Biochemical Analyses

Selected compounds of Krebs cycle and respiratory chain were determined in the hemolymph solutions and fat body supernatants:

- Acetyl Coenzyme A (acetyl-CoA) concentration according to the protocol provided by the manufacturer of the commercial kit Acetyl-CoA Carboxylase Microplate Assay Kit, MyBioSource, Inc., San Diego, CA, USA, no. MBS4504202;
- Isocitrate dehydrogenase (IDH) activity according to the protocol provided by the manufacturer of the commercial kit Human IDH2/Isocitrate dehydrogenase 2 ELISA Kit, Assay Genie Ltd., Dublin, Irland, no. HUFI01073;
- Alpha-Ketoglutarate (AKG) concentration according to the protocol provided by the manufacturer of the commercial kit Alpha-Ketoglutarate Assay Kit (Colorimetric), Cell Biolabs, Inc., San Diego, CA, USA, no. MET-5131;
- Succinate concentration according to the protocol provided by the manufacturer of the commercial kit Succinate Colorimetric Assay Kit, Sigma Aldrich, Schnelldorf, Germany, no. MAK184;
- Fumarate concentration according to the protocol provided by the manufacturer of the commercial kit Fumarate Assay Kit, Sigma Aldrich, Schnelldorf, Germany, no. MAK060;
- Nicotinamide adenine dinucleotide (NADH_2_) concentration according to the protocol provided by the manufacturer of the commercial kit Mouse nicotinamide adenine dinucleotide (NADH) ELISA Kit, MyBioSource, Inc., San Diego, CA, USA, no. MBS2602852;
- Cytochrome c Oxidase (COX) activity according to the protocol provided by the manufacturer of the commercial kit Cytochrome c Oxidase Assay Kit, Sigma Aldrich, Schnelldorf, Germany, no. CYTOCOX1-1KT;
- Cytochrome c reductase (UQCR) activity according to the protocol provided by the manufacturer of the commercial kit Cytochrome c Reductase (NADPH) Assay Kit, Sigma Aldrich, Schnelldorf, Germany, no. CY0100-1KT;
- Adenosine triphosphate (ATP) concentration according to the protocol provided by the manufacturer of the commercial kit ATP Assay Kit (Colorimetric/Fluorometric), Abcam, Cambridge, UK, no. ab83355.

Enzyme activities were expressed in U/mg protein. Protein concentrations [mg/mL] were determined using the Lowry method, which was modified by Schacterle and Pollack [55] and Łoś and Strachecka [54].

### 4.3. Statistical Analyses

The results were analyzed statistically using Statistica software, version 13.3 (2017) for Windows, StatSoft Inc., Tulsa, OK, USA. Data distribution was checked using the Shapiro–Wilk test. The effects of the tissue/fat body location (hemolymph and the fat body from tergite 3, tergite 5 and the sternite) in each age group (*n*  =  30 bees) on acetyl-CoA, AKG, succinate, fumarate, NADH_2_ and ATP concentrations and IDH, COX and UQCR activities were measured with the Kruskal–Wallis test. The effects of age (1-, 14-, 21-, 28- and 35-day-old) on acetyl-CoA, AKG, succinate, fumarate, NADH_2_ and ATP concentrations and IDH, COX and UQCR activities for the particular tissues/fat body locations (hemolymph and the fat body from tergite 3, tergite 5 and the sternite) were assessed in a similar fashion. For each tissue/fat body location (hemolymph and the fat body from tergite 3, tergite 5 and the sternite), acetyl-CoA, AKG, succinate, fumarate, NADH_2_ and ATP concentrations and IDH, COX and UQCR activities were compared between the age groups with the Mann–Whitney U test.

## 5. Conclusions

- This paper compares for the first time the concentrations/activities of the main components of the Krebs cycle and the respiratory chain in the hemolymph and fat body segments (tergites 3 and 5 and the sternite) of naturally and prematurely (affected by *V. destructor*) aging worker bees. The obtained results suggest that these components can be used as biomarkers of the aging process.
- The highest concentrations/activities of the tested compounds were detected in tergite 3. An analysis of the fat body segments will allow for a better understanding of the metabolic functions of the particular segments. This knowledge will enable an elucidation of the aging processes, mechanisms of immunity, adaptation to new conditions or resistance to various pollutants, e.g., pesticides.
- *V. destructor* causes a decrease in the concentrations/activities of acetyl-CoA, IDH, AKG, succinate, fumarate, NADH_2_, UQCR, COX and ATP. Consequently, *V. destructor* infestation of bees can lead to changes in their physiology and immunity, resulting in weakened bee colonies and premature aging of bees.
- One of the markers of aging in both bees and humans is changes in energy metabolism. Humans, like bees, age at different rates and times, therefore a comparison between younger (1-day-old) and older workers (28 and 35 days of age) will allow for understanding interindividual differences in the context of the aging process. In the future, research on changes in energy metabolism may help develop strategies to delay the aging process and improve human health.

## Data Availability

Data contained within the article. At a justified request of the interested party, they may be made available by the corresponding author.

## Supplementary Materials

The following supporting information can be downloaded at the following link: https://www.mdpi.com/article/10.3390/ijms26157294/s1.
