# Supplementary material for: The Efficiency of the Krebs Cycle and the Respiratory Chain in Physiologically and Prematurely Aging Bees (Apis mellifera)
Source: Int J Mol Sci. 2025 Jul 28;26(15):7294. doi: 10.3390/ijms26157294 (PMC12346989; doi:10.3390/ijms26157294)
Supplement: Supplementary file 1 [file ijms-26-07294-s001.zip › ijms-3745631-supplementary.pdf]

Table S1. Effect of tissue location: hemolymph, tergite 3, tergite 5 and sternite in the particular age groups on the concentrations of acetyl-CoA, AKG, succinate, fumarate, $NADH_2$, ATP and the activities of IDH, COX and UQCR in the naturally (physiologically) and prematurely (affected by *V. destructor*) aging workers.

	**Acetyl-CoA**	**IDH**	**AKG**	**Succinate**	**Fumarate**
Hemolymph	H = 47.97<br>p = 0.000	H = 131.25<br>p = 0.000	H = 130.22<br>p = 0.000	H = 141.78<br>p = 0.000	H = 137.35<br>p = 0.000
Tergite 3	H = 134.91<br>p = 0.000	H = 98.81<br>p = 0.000	H = 109.41<br>p = 0.000	H = 100.84<br>p = 0.000	H = 142.58<br>p = 0.000
Tergite 5	H = 168.17<br>p = 0.000	H = 111.96<br>p = 0.000	H = 141.89<br>p = 0.000	H = 142.49<br>p = 0.000	H = 142.58<br>p = 0.000
Sternite	H = 71.87<br>p = 0.000	H = 108.63<br>p = 0.000	H = 109.68<br>p = 0.000	H = 102.31<br>p = 0.000	H = 131.87<br>p = 0.000

	**$NADH_2$**	**COX**	**UQCR**	**ATP**
Hemolymph	H = 129.53<br>p = 0.000	H = 136.00<br>p = 0.000	H = 131.67<br>p = 0.000	H = 136.15<br>p = 0.000
Tergite 3	H = 80.63<br>p = 0.000	H = 142.58<br>p = 0.000	H = 131.78<br>p = 0.000	H = 109.68<br>p = 0.000
Tergite 5	H = 101.28<br>p = 0.000	H = 142.34<br>p = 0.000	H = 135.21<br>p = 0.000	H = 109.68<br>p = 0.000
Sternite	H = 85.41<br>p = 0.000	H = 142.59<br>p = 0.000	H = 133.02<br>p = 0.000	H = 118.56<br>p = 0.000

H — statistical value for the Kruskal–Wallis test; p—probability value.

Table S2. Effect of age: 1, 14, 21, 28 and 35 days old for individual tissues on the concentrations of acetyl-CoA, AKG, succinate, fumarate, $NADH_2$, ATP and the activities of IDH, COX and UQCR in the naturally (physiologically) and prematurely (affected by *V. destructor*) aging workers.

**Group**	**Age (Days)**	**Acetyl-CoA**	**IDH**	**AKG**	**Succinate**	**Fumarate**
control	1	H = 102.29<br>p = 0.000	H = 111.57<br>p = 0.000	H = 111.52<br>p = 0.000	H = 100.24<br>p = 0.000	H = 101.44<br>p = 0.000
control	14	H = 97.33<br>p = 0.000	H = 93.55<br>p = 0.000	H = 80.36<br>p = 0.000	H = 88.00<br>p = 0.000	H = 68.05<br>p = 0.000
V. destructor	14	H = 96.75<br>p = 0.000	H = 81.66<br>p = 0.000	H = 89.19<br>p = 0.000	H = 99.70<br>p = 0.000	H = 104.86<br>p = 0.000
control	21	H = 111.47<br>p = 0.000	H = 111.57<br>p = 0.000	H = 99.99<br>p = 0.000	H = 107.89<br>p = 0.000	H = 104.85<br>p = 0.000
V. destructor	21	H = 70.26<br>p = 0.000	H = 104.96<br>p = 0.000	H = 101.04<br>p = 0.000	H = 102.65<br>p = 0.000	H = 96.09<br>p = 0.000
control	28	H = 106.09<br>p = 0.000	H = 108.34<br>p = 0.000	H = 103.17<br>p = 0.000	H = 111.31<br>p = 0.000	H = 100.96<br>p = 0.000
control	35	H = 105.17<br>p = 0.000	H = 110.13<br>p = 0.000	H = 110.63<br>p = 0.000	H = 110.14<br>p = 0.000	H = 111.57<br>p = 0.000

**Group**	**Age (Days)**	**$NADH_2$**	**COX**	**UQCR**	**ATP**
control	1	H = 111.60<br>p = 0.000	H = 100.43<br>p = 0.106	H = 89.81<br>p = 0.000	H = 98.22<br>p = 0.000
control	14	H = 88.15<br>p = 0.000	H = 39.62<br>p = 0.000	H = 109.36<br>p = 0.000	H = 87.05<br>p = 0.000
V. destructor	14	H = 89.71<br>p = 0.000	H = 98.48<br>p = 0.000	H = 91.30<br>p = 0.000	H = 82.74<br>p = 0.000

control	21	H = 109.29<br>$p = 0.000$	H = 101.05<br>$p = 0.000$	H = 100.46<br>$p = 0.000$	H = 106.99<br>$p = 0.000$
V. destructor	21	H = 111.57<br>$p = 0.000$	H = 101.60<br>$p = 0.000$	H = 100.59<br>$p = 0.000$	H = 93.38<br>$p = 0.000$
control	28	H = 111.57<br>$p = 0.000$	H = 107.90<br>$p = 0.000$	H = 111.32<br>$p = 0.000$	H = 102.38<br>$p = 0.000$
control	35	H = 106.35<br>$p = 0.000$	H = 111.57<br>$p = 0.000$	H = 111.55<br>$p = 0.000$	H = 110.98<br>$p = 0.000$

H — statistical value for the Kruskal–Wallis test; p — probability value.
